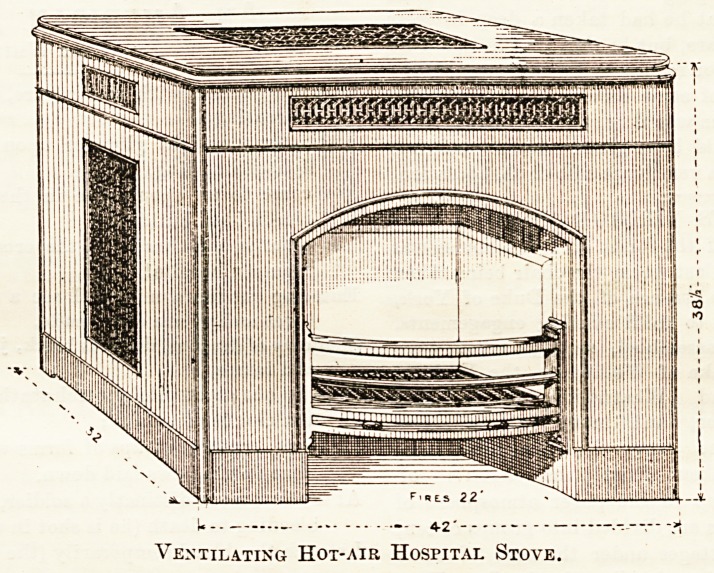# Heating and Ventilation

**Published:** 1895-04-13

**Authors:** 


### PRACTICAL DEPARTMENTS.

### HEATING AND VENTILATION.

Modern Fireplaces (*continued from page* 447, *Vol. XVII.*).

In our last article we spoke of the "Galton" warming and ventilatlng stoves, giving a sketch of one. Their object is to supply fresh warmed air into a room in place of that extracted by the chimney. The fresh air is admitted to a chamber behind the grate, where it becomes heated, and is thence carried by a flue to the upper part of the room or ward, where it mingles with the currents already existing. In this way a uniform temperature is easily maintained, no draughts interfere with comfort, and it has been shown by experiment that the amount of heat thus utilised is three times as much as in the case of an ordinary grate. The stove is of iron, except for the fire-clay cradle which holds the fire.

Ventilating Hot-air Hospital Stove.

These grates are also made in another form for placing in the centre of the ward, the chimney passing under the floor. The main body of the stove is a mass of fire-clay; in fact, every part of the stove "employed to warm the fresh air, with which the fire has direct contact, is of fire-clay." Sir Douglas Galton explains that this feature is of especial importance in hospitals, where purity of atmosphere is of such paramount necessity, owing to the fact that iron, and especially cast-iron, heated to a high temperature, admits of the passage through it of carbonic oxide from imperfect combustion of the fuel, and also that highly-heated iron in contact with air may "act on the organic matter, diminish the oxygen, and interfere with the freshness of the air." These stoves are in use at the Herbert Hospital, Woolwich. They are made by Messrs. Yates and Haywood and by Messrs. Kennard, both of Upper Thames Street.

Messrs. McDowall, Steven, and Co., also of Upper Thames Street, are large manufacturers of these and many other grates especially adapted for hospital and institution use. Notably we should mention their "ventilating hot-air stove," supplied with two fires, for the centre of a ward, of which we give a drawing, by permission of the makers. In this the fresh air can be brought from outside, passing out at the top and side of the stove without going through the fire.

The best method of warming and ventilating hospital wards is a vexed question, opinions differing widely with regard to the comparative advantages of open fires, hot-air stoves such as those just described, or entire systems of heating by hot water or air, and the abolition of the cheerful "English" fire altogether. It may be remembered that in the course of the recent alterations at the Chelsea Hospital for Women, the method of warming there in vogue was condemned, and the hot-air apparatus formerly in use has been consequently discarded in favour of open fires in the wards, and stoves for hall and passages. The stoves which have been put in for the latter purpose are made by Messrs. Hunt, of New Oxford Street, and appear to answer admirably, being economical both of fuel and labour in stoking, as stoves go, and at the same time giving out a good heat.

We have already mentioned Mr. Snell's thermhydric grate. Stoves on this principle, with double fires, are in use in the New Infirmary at Lewisham. Two of these with two fires each, and the accompanying hot-water coils, were found to warm the large wards completely, even through the past unusual winter, and in ordinary weather one fire in each stove is found to be quite sufficient.

It will be seen from the brief mention here made of the grates and stoves most in favour at the present time, that very considerable improvements have been effected of late years in heating appliances for large areas, and further improvements in more or less important particulars are being constantly brought out. In these days of competition such progress is inevitable, and if mistakes are made in the choice of apparatus for new buildings, the fault will be found to lie rather with those on whom the responsibility of choice rests than on any lack of suitable material.

## Figures and Tables

**Figure f1:**